# High-performance flexible transparent micro-supercapacitors from nanocomposite electrodes encapsulated with solution processed MoS_2_ nanosheets

**DOI:** 10.1080/14686996.2021.1978274

**Published:** 2021-10-13

**Authors:** Vivekanandan Raman, Dongjoon Rhee, Aravindha Raja Selvaraj, Jihyun Kim, Kandasamy Prabakar, Joohoon Kang, Han-Ki Kim

**Affiliations:** a School of Advanced Materials Science and Engineering, Sungkyunkwan University, Suwon, South Korea; b School of Electrical Engineering, Pusan National University, Busan, South Korea

**Keywords:** Micro-supercapacitor, flexible transparent electrode, nanocomposite, molybdenum disulfide, pseudocapacitance, 50 Energy Materials, 105 Low-Dimension (1D/2D) materials, 201 Electronics / Semiconductor / TCOs; 207 Fuel cells / Batteries / Super capacitors, 103 Composites

## Abstract

Two-dimensional molybdenum disulfide (MoS_2_) nanosheets have emerged as a promising material for transparent, flexible micro-supercapacitors, but their use in electrodes is hindered by their poor electrical conductivity and cycling stability because of restacking. In this paper, we report a novel electrode architecture to exploit electrochemical activity of MoS_2_ nanosheets. Electrochemically exfoliated MoS_2_ dispersion was spin coated on mesh-like silver networks encapsulated with a flexible conducting film exhibiting a pseudocapacitive behavior. MoS_2_ nanosheets were electrochemically active over the whole electrode surface and the conductive layer provided a pathway to transport electrons between the MoS_2_ and the electrolyte. As the result, the composite electrode achieved a large areal capacitance (89.44 mF cm^−2^ at 6 mA cm^−2^) and high energy and power densities (12.42 µWh cm^−2^ and *P* = 6043 µW cm^−2^ at 6 mA cm^−2^) in a symmetric cell configuration with 3 M KOH solution while exhibiting a high optical transmittance of ~80%. Because the system was stable against mechanical bending and charge/discharge cycles, a flexible micro-supercapacitor that can power electronics at different bending states was realized.

## Graphical abstract

**Figure:**
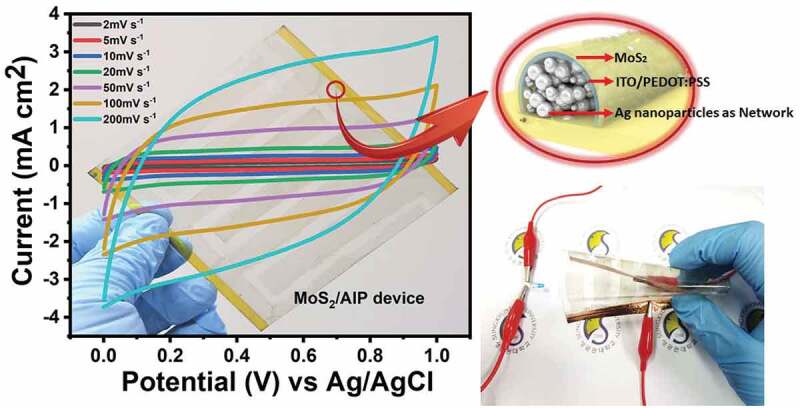

## 1. Introduction

Transparent, wearable electronics are critical for realizing displays, sensors, and implants that can be seamlessly integrated into human body or arbitrary surfaces [1–3]. As a power source to operate these devices, miniaturized electrochemical energy storage devices with high optical transmittance and mechanical flexibility are in strong demand [4,5]. In particular, micro-supercapacitors have gained significant interests for applications where long cycle life, fast charge-discharge capability, and high power density are desired such as implantable biochips and sensors [6–8]. Most work on supercapacitors have used porous carbon film electrodes, where charges are stored in electric double layers that result from electrostatic adsorption of electrolyte ions on the surface [9,10]. Although a high surface area (> 2000 m^2^ g^−1^) can be obtained, the areal capacitance is mostly less than 3 mF cm^−2^ and thus the energy and power densities are limited [11–13]. To enhance the electrochemical performance of the supercapacitors, thin films of pseudocapacitive transition metal oxides such as RuO_2_ and MnO_2_ have been widely employed [14–16]. These materials can store additional charges through fast and reversible redox reactions at or near the surface, which results in a significant increase in capacitance compared to electrodes only exploiting electric double layers [17]. Fabricating flexible transparent supercapacitors based on these films, however, is challenging because they are typically brittle and opaque [6]. Although decreasing the film thickness can increase the mechanical flexibility and optical transmittance, such strategy typically leads to decreased energy and power densities. Alternatively, transparent conducting polymer films can be used, but their cycling stability is limited because of swelling during charge/discharge processes [18–21].

Two-dimensional (2D) nanomaterials are emerging as a candidate for wearable electrochemical energy storage devices [22,23]. For example, graphene features high specific surface area, electrical conductivity, optical transparency, and high mechanical strength essential for creating flexible transparent supercapacitors [24–26]. Graphene, however, can only store charges by the formation of electric double layers, which motivates investigation of 2D transition metal dichalcogenides with a pseudocapacitive behavior for achieving higher areal capacitance and energy density [27,28]. In particular, MoS_2_ nanosheets offer a rich charge storage mechanisms including generation of electric double layers in inter- and intra-sheet regions and faradaic charge transfer processes involved with oxidation/reduction of the Mo atom [29]. One drawback of using MoS_2_ nanosheets in supercapacitors is their low electrical conductivity compared to graphene [30,31]. Furthermore, the self-assembled nanosheets restacking reduces the surface area accessible for charge storage resulting in the lower charge/discharge cyclic stability [32,33]. Challenges in using MoS_2_ as a stand-alone electrode material have been overcome by integrating the nanosheets with conductive nanomaterials [33–37]. For example, a transparent electrode based on MoS_2_-decorated silver nanowire networks demonstrates enhanced electrochemical activity compared to MoS_2_ films because electron transport can be facilitated by the silver core [34]. The resulting supercapacitor is stable against repeated charge/discharge and bending cycles because restacking of the nanosheets is prevented by the robust interfacial adhesion between silver and MoS_2_. However, this electrode architecture is still limited by a high fraction of inactive areas that are not covered by the nanowires. Coating the entire surface with a conductive layer and MoS_2_ nanosheets could increase the areal capacitance [38], but simultaneously achieving the mechanical flexibility and optical transparency is difficult.

Herein we present a scalable method to couple MoS_2_ nanosheets with conductive metal nanostructures for realizing flexible transparent micro-supercapacitors. Mesh-like networks of silver nanoparticles were first encapsulated with a flexible conducting film consisting of indium tin oxide nanoparticles and poly(3,4-ethylenedioxythiophene) polystyrene sulfonate (ITO/PEDOT:PSS), followed by spin coating of electrochemically exfoliated MoS_2_ nanosheets. The resulting electrode exhibits a high optical transmittance (~80%) and a large areal capacitance (89.44 mF cm^−2^ at 6 mA cm^−2^) in a symmetric cell configuration with 3 M KOH solution. Significantly, the energy and power densities (12.42 µWh cm^−2^ and 6043 µW cm^−2^ at 6 mA cm^−2^) were much higher compared to previously reported flexible transparent micro-supercapacitors. The enhanced electrochemical performance was possible because (1) the entire surface of the electrode was active for charge storage *via* electric double layer formation and faradaic charge transfer on both MoS_2_ nanosheets and ITO/PEDOT:PSS surface and (2) the ITO/PEDOT:PSS layer functioned as a good electrical conductor between silver networks and MoS_2_ nanosheets. The system was stable against extensive cycles of mechanical bending (less than 2% change in resistance after 10,000 cycles) and charge/discharge processes (6% loss in capacity after 6000 cycles at 8 mA cm^−2^). Taking advantage of the composite electrode, we demonstrated a solid-state micro-supercapacitor that can power electronics at different bending states.

## 2. Experimental

### 2.1. Synthesis of MoS_2_

Ultrathin MoS_2_ nanosheets were produced by the electrochemical exfoliation. An MoS_2_ crystal (purchased from HQ graphene) was fixed with an alligator clip as a cathode and a graphite rod as a counter electrode. As an intercalant for exfoliation, tetra-heptyl ammonium bromide was dissolved in acetonitrile at a concentration of 5 mg/mL. The electrochemical reaction was conducted with an applied voltage of 7 V for 1 hour. After the reaction, the MoS_2_ crystal was rinsed with ethanol and sonicated in 0.2 M polyvinylpyrrolidone in dimethylformamide (DMF) solution for 30 minutes. To remove unexfoliated crystals, the as-prepared dispersion was centrifuged at 4000 rpm for 10 minutes. DMF was then exchanged with isopropanol for spin coating [39].

### 2.2. Fabrication of the mesh-like Ag network

Ag networks were formed on flexible polyethylene terephthalate (PET) substrates using a bar-coating system (KP-3000VH, Kipae E&T Co.). The Ag nanoparticle solution was first made by mixing 54 wt% of TCC A with 46 wt% of TC3C B solution and sonicated three times for 30 seconds for homogenization. To increase the wettability of the PET substrate, we first decreased the surface energy by applying a surface treatment (DOF Co.). The Ag nanoparticle solution was then drop casted onto the pre-treated PET substrate and bar-coated at a speed of 30 mm/s. The film was dried at 50°C for 60 seconds and then at 150°C for 90 seconds to produce self-assembled embossed-type Ag network films.

### 2.3. Deposition of the ITO/PEDOT:PSS nanocomposite layer on the Ag-coated PET substrate

First, indium nitrate (In(NO_3_)_3_) and tin chloride (SnCl_2_) precursor solutions were prepared by mixing In metal with HNO_3_ solution and Sn metal with HCl solution. The precursor solutions were then mixed at a stoichiometric ratio of In(NO_3_)_3_:SnCl_2_ = 90:10, followed by addition of ammonium hydroxide (NH_4_OH) solution to form indium tin hydroxide precipitates. The NH_4_OH solution was added until the pH became 9.75, which was essential for achieving uniformity in the nanoparticle size. The resulting precipitates were dried at 80°C and sintered in a nitrogen atmosphere to form indium tin oxide (ITO) nanoparticles. Then, the nanoparticles were thoroughly grinded using water as a dispersion medium at a concentration of 30 wt%. After grinding, the solution was dispersed in 70 wt% water. A stable dispersion of ITO nanoparticles was achieved without any particle agglomeration by using a vibrating miller with 0.2 mm zirconia beads for 30 minutes. The dispersed ITO nanoparticles were mixed with an aqueous PEDOT:PSS solution at a 1:1 mass ratio. An even dispersion of ITO and PEDOT:PSS was obtained by mixing the solution with the vibrating miller using 0.2 mm zirconia beads. Next, the PET substrate (~10 × 10 cm^2^) coated with Ag networks was treated with UV ozone for 10 minutes for surface activation. Then, the ITO/PEDOT:PSS nanocomposite layer was spin coated at 3000 rpm for 40 seconds three times, followed by drying at 150 °C for 10 minutes to evaporate the solvent. The resulting ITO/PEDOT:PSS film was post-treated with 1 M H_2_SO_4_ at 160 °C for 5 minutes, washed thoroughly with distilled water, and dried at 150 °C for 5 minutes. After repeating this post-treatment process five times, the ITO/PEDOT:PSS layer was annealed with O_2_ plasma to crystallize the ITO nanoparticles based on a method described in our previous findings [40].

### 2.4. Fabrication of the MoS_2_/AIP electrode

The ITO/PEDOT:PSS-coated Ag network electrode (AIP electrode) was exposed to UV ozone for 10 minutes for activating the surface. Then, the MoS_2_ nanosheet solution was spin coated on the electrode at 1500 rpm for 30 seconds and dried at 150°C for 10 minutes to create the MoS_2_-decorated AIP electrode (MoS_2_/AIP electrode).

### 2.5. Fabrication of solid-state micro-supercapacitors

First, MoS_2_ nanosheets were deposited on the AIP electrode through a mask. A gel electrolyte based on polyvinyl alcohol (PVA) was prepared by dissolving 1 g of PVA in deionized water at 80°C for 2 hours and adding 1 g of KOH (10 mL) with a constant stirring until the mixture became a gel type solution. Then, the PVA-KOH gel was spin coated on the electrode at 1000 rpm for 40 seconds, followed by drying at room temperature and mask removal. Silver paste and copper tape were attached to the areas that were not masked during spin coating to form electrical contacts.

### 2.6. Material characterization

The electrical properties of the MoS_2_/AIP electrode were characterized using Hall measurements (HL5500PC, Accent optical technology, US). The optical properties of the thin film electrodes were measured using UV/Visible spectrometer (V-670, Jasco), US. The surface morphology MoS_2_/AIP electrode was analyzed using FE-SEM, Hitachi, Japan (field-emission scanning electron microscope). Crystal structure and chemical composition of samples were examined using XRD (x-ray diffraction), Rigaku, Japan and XPS (x-ray photoelectron spectroscopy), Thermo Fisher, US. The electrochemical behavior of electrodes was evaluated using Bio Logic SP-150, Bio Logic, France instrument. For the three-electrode measurements, Ag/AgCl and Pt wires were used as reference and counter electrodes, respectively. 3 M KOH aqueous solution was used as an electrolyte.

## 3. Results and discussion

**Figure 1.**
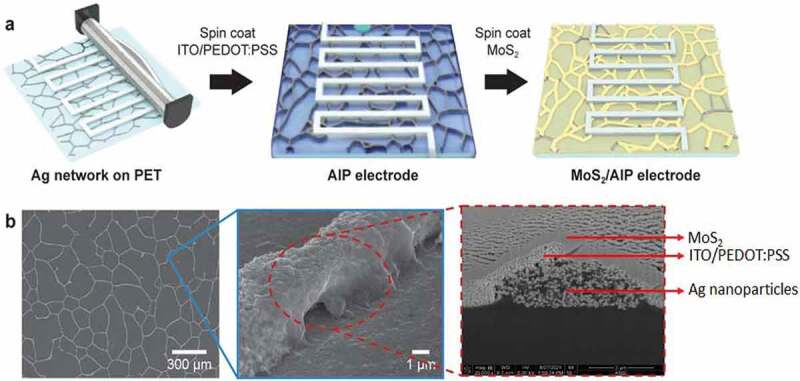
MoS_2_/AIP electrode for transparent flexible micro-supercapacitors. (a) Schematic illustration of the fabrication process. (b) Top-view and cross-sectional SEM images of the nanocomposites consisting of Ag nanoparticles, ITO/PEDOT:PSS layer, and MoS_2_ nanosheets

Figure 1(a) describes the method to create MoS_2_/AIP electrode based on solution processing techniques. First, Ag nanoparticle solution was bar coated on a polyethylene terephthalate (PET) substrate through a shadow mask with interdigitated patterns (figure width and length were 0.5 cm and 8 cm, respectively). Ag nanoparticles self-assembled into mesh-like networks during drying in regions that were not covered by the mask. After a UVO treatment for activating the surface, an aqueous solution containing ITO nanoparticles and PEDOT:PSS was spin coated, followed by a

H_2_SO_4_ post-treatment process and plasma annealing to crystallize ITO without affecting PEDOT:PSS. We denote the PET substrate decorated with the Ag networks and ITO/PEDOT:PSS layer as AIP electrode because the AIP composite serves as a current collector for micro-supercapacitors. 2 H-phase MoS_2_ nanosheets from electrochemical exfoliation (Figure S1) were then spin coated onto the AIP electrode as an active material. Based on scanning electron microscope (SEM) images, we confirmed the cross-section of MoS_2_/AIP electrode coating layers (Figure 1(b)).

**Figure 2.**
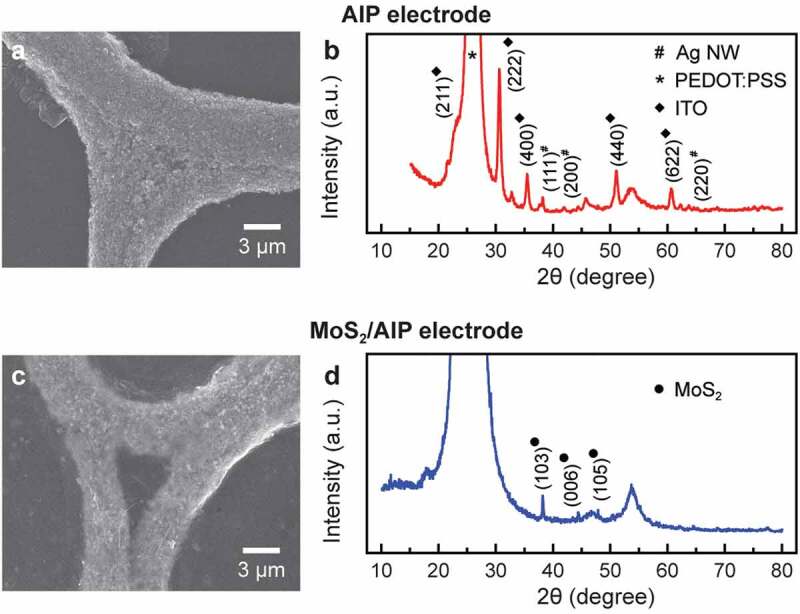
Structural characteristics of electrodes before and after coating MoS_2_ nanosheets. (a) SEM image and (b) XRD spectrum of AIP electrode. (c) SEM image and (d) XRD spectrum of MoS_2_/AIP electrode

To reveal structural characteristics of the MoS_2_-coated AIP electrode (MoS_2_/AIP electrode) in more detail, we investigated samples before and after casting MoS_2_ nanosheets using SEM and x-ray diffraction (XRD) analysis (Figure 2). High resolution SEM images of the AIP shows that individual Ag nanoparticles maintained their morphology after ITO/PEDOT:PSS coating and plasma annealing (Figure 2(a)).

The presence of Ag nanoparticles was further confirmed by diffraction peaks at 2θ = 38.2º, 44.2º and 64.5º corresponding to (111), (200), and (220) planes of silver (Figure 2(b)) [41]. Peaks at 2θ = 21.5º, 30.6º, 35.5º, 51.1º, and 60.7º can be assigned to (211), (222), (400), (440), and (622) reflections from polycrystalline cubic-phase ITO, which resulted from the second-order phase transition of amorphous ITO after plasma annealing, while the high-intensity peak at 2θ = 25.2º corresponds to PEDOT:PSS [40]. When MoS_2_ dispersion were spin coated on the AIP electrode, the MoS_2_ nanosheets evenly encapsulated the network structure because of strong electrostatic attractions (Figure 2(c)). The MoS_2_/AIP electrode was characterized by XRD peaks at 2θ = 38.2º, 44.4º, and 47.9º, which correspond to (103), (006), and (105) reflections from MoS_2_ in the 2 H phase (Figure 2(d)) [42].

**Figure 3.**
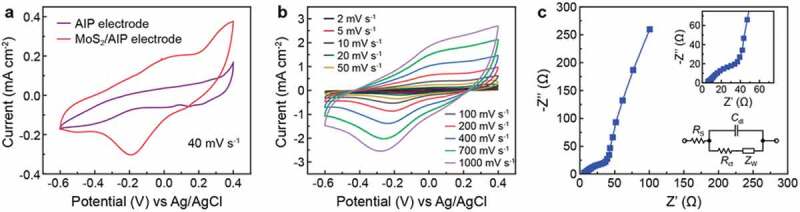
Electrochemical characterization of the MoS_2_/AIP electrode based on a three-electrode set up. (a) Cyclic voltammograms of AIP and MoS_2_/AIP electrodes (scan rate: 40 mV s^−1^). (b) Cyclic voltammograms of the MoS_2_/AIP under different scan rates. (c) Electrochemical impedance spectroscopy of the MoS_2_/AIP electrode

**Figure 4.**
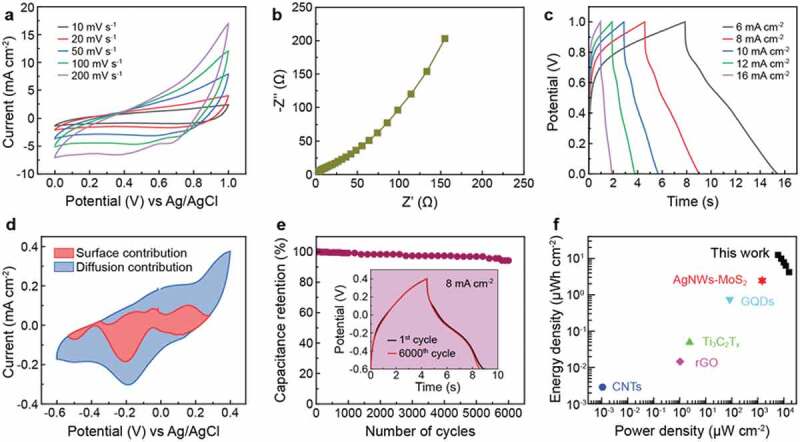
Electrochemical behavior of the MoS_2_/AIP electrode in a symmetric two electrode configuration. (a) Cyclic voltammograms at different scan rates. (b) Nyquist plot from electrochemical impedance spectroscopy. (c) Galvanostatic charge/discharge curves at different currents. (d) Deconvolution of charge contributions to the capacitance of the AIP and MoS_2_/AIP electrodes at 40 mV s^−1^ (e) Cyclic stability of the MoS_2_/AIP electrode. (f) Ragone plot comparing energy and power densities of the MoS_2_/AIP electrode and other systems. Reference data adapted with permission from ref [26], copyright 2018 American Chemical Society; ref [34], copyright 2019 Elsevier; ref [52], copyright 2017 Wiley; ref [53], copyright 2014 Nature Publishing Group; ref [54], copyright 2016 Elsevier

The electrochemical activity was first evaluated by performing cyclic voltammetry (CV) of AIP and MoS_2_/AIP electrodes (1 × 1 cm^2^) using three-electrode configuration (Figure 3(a)). Among different aqueous electrolytes, we focused on KOH because we found a stable performance with good electrochemical behavior compared to other electrolytes (Figure S2). The Figure 4(a) shows the electrode performance at 20 mV S^−1^ with Na_2_SO_4_ and H_2_SO_4_. These electrolytes tend show lower current value with abnormal redox behavior. The integral area of the CV curve increases for the MoS_2_/AIP electrode due to the presences of ITO/PEDOT:PSS composite in Ag network as the current collector. It facilitates by improving the electrochemical performance and boost the redox behaviour as PEDOT:PSS encourages and enhance the performance of the electrode [43,44]. The MoS_2_/AIP electrode CV curve was nearly rectangular, which is indicative of capacitive behavior [45]. Deviation from an ideal rectangle suggests that MoS_2_/Ag network based ITO/PEDOT:PSS layer not only exhibits electrical double-layer capacitance but also shows pseudocapacitance involved with faradaic charge transfer reactions [46,47]. AIP as the current collector enhanced the electrochemical performance of the system with significantly increased current and integral area of CV curve. We attribute this enhanced charge storage formed due to the redox behavior of the MoS_2_ nanosheets and AIP current collector. Moreover, MoS_2_/AIP electrode in KOH electrolyte at high scan rate promotes protons for the surface redox charge transfer due to its thin and small ion size compared to the other electrolytes (Figure S2). The MoS_2_/AIP electrode retained strong redox peaks even at higher scan rates (up to 1000 mV s^−1^) denoting the pseudocapacitive behavior (Figure 3(b)). Electrochemical characteristics of the system was further investigated by electrochemical impedance spectroscopy (Figure 3(c)). We analyzed the data based on the Randles equivalent circuit [48], which models the electrode/electrolyte system with an electrolyte resistance (*R*_S_) connected in series with the double layer capacitance (*C*_dl_) that is in parallel with the charge transfer resistance (*R*_ct_) and Warburg impedance (*Z*_W_) (Figure 3(c), inset). From the Nyquist plot, we determined that the charge transfer resistance of the MoS_2_/AIP electrode was approximately 25 Ω. The curve reveals capacitive characteristics with fast current reaction with low resistivity. In the low frequency range, the impedance spectrum followed a nearly vertical line because of surface ion diffusion.

The electrochemical performance of the symmetric MoS_2_/AIP hetero structured electrode was investigated via two electrode system. The 1 × 1 cm electrode was assembled as the positive and negative electrode submerged in 3 M KOH electrolyte. The CV curves of the symmetric electrode cells (Figure 4(a)) exhibited a similar quasi-rectangular shape similar to the data measured from the three-electrode set up (Figure 3(a,b)). The system showed a good electrochemical stability over a potential window from 0 V to 1 V when compared with other potential windows (Figure S3) except that the CV curves were bit constrained with diffusion of ion on the surface of the electrode under increasing scan rates. MoS_2_/AIP composite electrode performed better with KOH electrolyte when compared with Na_2_SO_4_ and H_2_SO_4_ electrolyte (Figure S4, S5).

Moreover, electrochemical impedance spectroscopy suggests that the charge transfer resistance was extremely low (~15.6 Ω) (Figure 4(b)). The lower-frequency regime in the Nyquist plot almost showed a vertical line, signifying the low resistive performance of the electrode. The electrochemical behaviour of the system was further analysed with galvanostatic charge/discharge (GCD) curves measured at various current densities (6–16 mA cm^−2^) over a potential window from 0 V to 1 V (Figure 4(c)). The slight drop at the beginning of the discharge cycle can be attributed to the internal resistance between the electrode and electrolyte. As the current increased, the resistivity between the electrolyte and electrode reduced, which decreased the contact and internal resistance. From the GCD data, we could calculate the capacitance of the MoS_2_/AIP electrode. Because the thickness and weight of the active material in our system was almost negligible for determining the gravimetric and volumetric capacitance [49,50], we estimated the areal capacitance using the equation [51]

(1) $$C=\frac{2\times I\times t}{V\times S}$$

where *C* denotes the areal capacitance, *I* is the discharge current, *t* is the discharge time (s), *V* is the potential window, and *S* is the area of the active material. The areal capacitance of the electrode was 89.44 mF cm^−2^ at a current density of 6 mA cm^−2^, which is much higher than that of a previously reported micro-supercapacitor consisting of a Ag nanowire current collector and MoS_2_ nanosheets (27.6 mF cm^−2^ at 0.2 V s^−1^) [34].

We further analysed the GCD data of the three electrode system to reveal surface contribution (*C*_s_, closely related to the electrical double layer capacitance) and diffusion contribution (*C*_d_, closely associated with the pseudocapacitance) to the capacitance of the MoS_2_/AIP electrode based on the Dunn’s method [52]. The current can be expressed as a combination of surface and diffusion components

(2) $$i\left(V\right)=k_{1}\upsilon +k_{2}\upsilon^{1/2}$$

where *i* (*V*) is the current at a given potential *V, v* is the scan rate, *k*_1_ and *k*_2_ are constants. By plotting *i* (*V*)/*v*^1/2^ against *v*^1/2^, we could obtain *k*_1_ and *k*_2_ from the slope and *y*-intercept. The surface (*k*_1_ *v*) and diffusion (*k*_2_ *v*^1/2^) contributions at each voltage were then be determined (Figure 4(d)). Within the potential window from −0.6 V to 0.4 V, we found that relative contributions of surface and diffusion contribution were 29.8% and 70.2% respectively, which implies that the MoS_2_/AIP electrode can store charges by showing the properties of electric double layers capacitor and as well as via faradaic charge transfer processes thus implying positively pseudocapacitive behaviour.

Measurement of the areal capacitance under repeated charging and discharging allows for evaluating the cyclic stability of the electrode (Figure 4(e)). Even after 6000 charge/discharge cycles at 8 mA cm^−2^, the MoS_2_/AIP-based micro-supercapacitor exhibited a nearly identical curve with a minor reduction in the discharge time (~0.3 s) compared with the 1^st^ cycle (Figure 4(e), inset). The capacity retention was about 94%, which highlights excellent cyclic stability of our system. The electrochemical performance was further characterized by quantifying the areal energy and power densities based on the following equations [53]:

(3) $$E=\frac{\frac{1}{2}CV^{2}}{3600}$$

(4) $$P=\frac{E}{t}\times 3600$$

where *E* and *P* are energy and power densities. The MoS_2_/AIP composite showed much higher energy and power densities (e.g. *E* = 12.423 µWh cm^−2^ and *P* = 6043 µW cm^−2^ at 6 mA cm^−2^) compared to many other flexible transparent micro-supercapacitor electrodes that were previously reported including electrodes based on Ag nanowire/MoS_2_ composites [34], 2D titanium carbide (MXene) films [54], carbon nanotubes (CNTs) [55], graphene quantum dots (GQDs) [56], reduced graphene oxide (rGO) sheets [26], and Co_3_O_4_ nanocrystals [57] (Figure 4(f) and Table 1).

**Table 1.** Transmittance and electrochemical performance of various flexible micro-supercapacitor electrodes

Electrode materials	Transmittance at 550 nm	Specific capacitance	Energy density	Power density	Ref.
AgNWs-MoS_2_	77.5%	27.6 mF cm^−2^ at 200 mV s^−1^	2.453 µWh cm^−2^	1472 μW cm^−2^	[34]
Ti_3_C_2_T_x_	72%	1.6 mF cm^−2^	0.05 μWh cm^−2^	2.4 μW cm^−2^	[54]
CNTs	75%	8.76 µF cm^−2^ at 0.3 µA cm^−2^	2.88 × 10^−3^ µWh cm^−2^	1.08 × 10^−3^ μW cm^−2^	[55]
GQDs	92.97%	9.09 μF cm^−2^ at 0.02 µA cm^−2^	0.727 µWh cm^−2^	83.4 μW cm^−2^	[56]
rGO	61%	208 μF cm^−2^ at 3 µA cm^−2^	1.45 × 10^−2^ µWh cm^−2^	1.04 μW cm^−2^	[26]
Co_3_O_4_	51%	6.03 mF cm^−2^ at 1 mV s^−1^	3.01 Wh kg^−1^	31 W kg^−1^	[57]
MoS_2_/AIP	79.56%	89.44 mF cm^−2^ at 6 mA cm^−2^	12.784 µWh cm^−2^	6043 μW cm^−2^	This work

**Figure 5.**
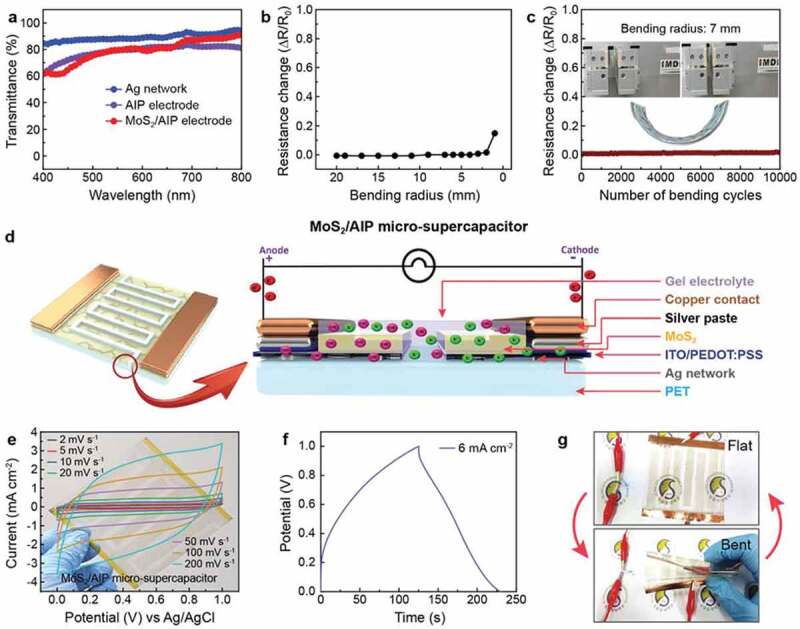
Transparent, flexible micro-supercapacitor based on the MoS_2_/AIP electrode. (a) Transmittance spectra of electrodes. Change in the electrical resistance of the MoS_2_/AIP electrode (b) at different bending radii and (c) during 10,000 cycles of mechanical bending up to a bending radius of 7 mm. (d) Schematic illustration of the MoS_2_/AIP micro-supercapacitor. (e) Cyclic voltammograms and (f) galvanostatic charge/discharge curve of the MoS_2_/AIP micro-supercapacitor. (g) Operation of a light emitting diode connected to the MoS_2_/AIP micro-supercapacitor at different bending states

Besides the electrochemical behavior, optical properties and mechanical robustness of the electrode are critical factors for realizing transparent flexible micro-supercapacitors. We first investigated the optical transmittance of the electrode after coating of each layer to clarify the effect of each component in the MoS_2_/AIP composite (Figure 5(a)). Ag networks on the PET substrate showed an excellent transmittance (*T*) of 88.16% at 550 nm, which slightly decreased to 79.36% (AIP electrode) after depositing the ITO/PEDOT:PSS layer. Significantly, the transmittance of the MoS_2_/AIP electrode (79.56%) was approximately the same as the AIP current collector because MoS_2_ nanosheets had only a minor impact on the optical transparency of the system. In addition, the AIP and MoS_2_/AIP electrodes exhibited low sheet resistances (*R*_S_) of 5.05 Ω/□ and 9.20 Ω/□, respectively. As a result, these transparent conducting electrodes achieved figures of merit (FoM = *T*^10^/*R*_S_) [58] significantly higher than other systems based on Ag nanocomposites (Table 2) [44,50,59]. The MoS_2_/AIP electrode also showed an excellent mechanical stability under bending test. The resistance did not change under bending up to bending radius of 2 mm although there was a slight increase under higher bending (bending radius < 2 mm), which might be attributed to breaking of the interlinked AIP current collector encapsulated by MoS_2_ (Figure 5(b)). Even after 10,000 cycles of bending up to a bending radius of 7 mm, the electrode showed a minimal resistance change (< 2%) (Figure 5(c)). To further highlight the potential of the MoS_2_/AIP electrode as a transparent flexible micro-supercapacitor, we fabricated a device consisting of two symmetric MoS_2_/AIP electrodes with an active area of ~6 cm^2^ and separated by a PVA-KOH gel electrolyte (Figure 5(d)). From the cyclic voltammetry analysis at different scan rates (2–200 mV s^−1^), we confirmed that the system functioned as a supercapacitor with a good electrochemical stability (Figure 5(e)).

**Table 2.** Electrical and optical properties of various transparent conducting electrodes

	Sheet resistance (Ohm/sq.]	Resistivity (×10^−3^ Ohm-cm)	Mobility (cm^2^/Vs)	Transmittance at 550 nm (%)	FoM (×10^−3^)	Ref.
Bare Ag network	12.61	6.3	4.49	88.16	22.92	This work
AIP electrode	5.05	1.01	1.44	79.36	39.77	This work
MoS_2_/AIP electrode	9.20	4.21	2.74	79.56	31.8	This work
Ag nanowire/PEDOT:PSS	25	-	-	85	7.874	[44]
Ag network/PTFE	11.64	-	-	80.20	9.45	[59]
Ag network(emboss type)	12.50	-	-	86.5	18.76	[50]

Also, the galvanostatic charge/discharge curve (6 mA cm^−2^) was approximately triangular with a low internal resistance (Figure 5(f)) and it was further confirmed in impedance spectroscopy revealing low resistivity as shown in (Figure S6) The areal capacitance of the device was 207 mF cm^−2^ with the energy density of 28.78 µWh cm^−2^ and power density of 1.03 mW cm^−2^ at 6 mA cm^−2^. As a proof-of-concept demonstration, we tested operating a light emitting diode (LED) using a charged MoS_2_/AIP micro-supercapacitor. The MoS_2_/AIP device could turn on the LED both at flat and bent states with a similar brightness. The degradation of the AIP and MoS_2_ electrodes are being studied via hall measurement as shown in Figure S7. The results showed that there is very minimal change in the sheet resistance which is negligible confirming that there is no degradation of the electrodes by oxidation or chemical degradation.

## 4. Conclusion

In summary, we have developed a method to create high-performance, flexible transparent micro-supercapacitors based on MoS_2_ nanosheets. By carefully designing the electrode architecture, we could effectively exploit electrochemical activity of the MoS_2_ nanosheets and achieve large areal capacitance and high energy and power densities. Moreover, the composite electrode exhibited outstanding optical transparency and stability against mechanical bending and charge/discharge cycles. Because all the components in our electrode could be formed using scalable, solution-based processes, we expect that our work offers a cost-effective means to fabricate portable energy storage devices for transparent wearable electronics.

## References

[1] Trung TQ, Lee NE. Materials and devices for transparent stretchable electronics. J Mater Chem C. 2017;5:2202–2222.

[2] Xu K, Lu Y, Takei K. Multifunctional skin-inspired flexible sensor systems for wearable electronics. Adv Mater Technol. 2019;4:1–25.

[3] Fernandes DF, Majidi C, Tavakoli M. Digitally printed stretchable electronics: a review. J Mater Chem C. 2019;7:14035–14068.

[4] Wang X, Lu X, Liu B, et al. Flexible energy-storage devices: design consideration and recent progress. Adv Mater. 2014;26:4763–4782. 24913891 10.1002/adma.201400910

[5] Liu W, Song MS, Kong B, et al. Flexible and stretchable energy storage: recent advances and future perspectives. Adv Mater. 2017;29:1603436. 10.1002/adma.201603436 28042889

[6] Zhao W, Jiang M, Wang W, et al. Flexible transparent supercapacitors: materials and devices. Adv Funct Mater. 2021;31:1–30.

[7] Jia R, Shen G, Qu F, et al. Flexible on-chip micro-supercapacitors: efficient power units for wearable electronics. Energy Storage Mater. 2020;27:169–186.

[8] García Núñez C, Manjakkal L, Dahiya R. Energy autonomous electronic skin. npj Flex Electron. 2019;3:1.

[9] Simon P, Gogotsi Y. Materials for electrochemical capacitors. Nat Mater. 2008;7:845. 18956000 10.1038/nmat2297

[10] Yu Z, Tetard L, Zhai L, et al. Supercapacitor electrode materials: nanostructures from 0 to 3 dimensions. Energy Environ Sci. 2015;8:702–730.

[11] Gamby J, Taberna PL, Simon P, et al. Studies and characterisations of various activated carbons used for carbon/carbon supercapacitors. J Power Sources. 2001;101:109–116.

[12] Pech D, Brunet M, Taberna PL, et al. Elaboration of a microstructured inkjet-printed carbon electrochemical capacitor. J Power Sources. 2010;195:1266–1269.

[13] Pech D, Brunet M, Durou H, et al. Ultrahigh-power micrometre-sized supercapacitors based on onion-like carbon. Nat Nanotechnol. 2010;5:651–654. 20711179 10.1038/nnano.2010.162

[14] Ardizzone S, Fregonara G, Trasatti S. “Inner” and “outer” active surface of RuO2 electrodes. Electrochim Acta. 1990;35:263–267.

[15] Zheng JP, Jow TR. A new charge storage mechanism for electrochemical capacitors. J Electrochem Soc. 1995;142(1):L6–8.

[16] Kuo S-L, Wu N-L. Investigation of pseudocapacitive charge-storage reaction of MnO[sub 2]⋅nH[sub 2]O supercapacitors in aqueous electrolytes. J Electrochem Soc. 2006;153:A1317.

[17] Zheng JP, Cygan PJ, Jow TR. Hydrous ruthenium oxide as an electrode material for electrochemical capacitors. J Electrochem Soc. 1995;142:2699–2703.

[18] Snook GA, Kao P, Best AS. Conducting-polymer-based supercapacitor devices and electrodes. J Power Sources. 2011;196:1–12.

[19] Zhao Q, Wang G, Yan K, et al. Binder-free porous PEDOT electrodes for flexible supercapacitors. J Appl Polym Sci. 2015;132:1–9.25866416

[20] Song J, Ma G, Qin F, et al. High-conductivity, flexible and transparent PEDOT:PSS electrodes for high performance semi-transparent supercapacitors. Polymers (Basel). 2020;12:450. 10.3390/polym12020450 PMC7077632 32075032

[21] Österholm AM, Shen DE, Dyer AL, et al. Optimization of PEDOT films in ionic liquid supercapacitors: demonstration as a power source for polymer electrochromic devices. ACS Appl Mater Interfaces. 2013;5:13432–13440. 24328278 10.1021/am4043454

[22] Yi F, Ren H, Shan J, et al. Wearable energy sources based on 2D materials. Chem Soc Rev. 2018;47:3152–3188. 29412208 10.1039/c7cs00849j

[23] Lin L, Chen J, Liu D, et al. Engineering 2D materials: a viable pathway for improved electrochemical energy storage. Adv Energy Mater. 2020;10:2002621.

[24] Yu A, Roes I, Davies A, et al. Ultrathin, transparent, and flexible graphene films for supercapacitor application. Appl Phys Lett. 2010;96:253105.

[25] Xu P, Kang J, Choi J-B, et al. Laminated ultrathin chemical vapor deposition graphene films based stretchable and transparent high-rate supercapacitor. ACS Nano. 2014;8:9437–9445. 25144124 10.1021/nn503570j

[26] Aytug T, Rager MS, Higgins W, et al. Vacuum-assisted low-temperature synthesis of reduced graphene oxide thin-film electrodes for high-performance transparent and flexible all-solid-state supercapacitors. ACS Appl Mater Interfaces. 2018;10:11008–11017. 29528215 10.1021/acsami.8b01938

[27] Cong X, Cheng C, Liao Y, et al. Intrinsic charge storage capability of transition metal dichalcogenides as pseudocapacitor electrodes. J Phys Chem C. 2015;119:20864–20870.

[28] Yu X, Yun S, Yeon JS, et al. Emergent pseudocapacitance of 2D nanomaterials. Adv Energy Mater. 2018;8:1702930.

[29] Joseph N, Shafi PM, Bose AC. Recent advances in 2D-MoS2and its composite nanostructures for supercapacitor electrode application. Energy Fuels. 2020;34:6558–6597.

[30] Acerce M, Voiry D, Chhowalla M. Metallic 1T phase MoS2 nanosheets as supercapacitor electrode materials. Nat Nanotechnol. 2015;10:313–318. 25799518 10.1038/nnano.2015.40

[31] Restrepo OD, Krymowski KE, Goldberger J, et al. A first principles method to simulate electron mobilities in 2D materials. New J Phys. 2014;16:105009.

[32] Wang S, Zhu J, Shao Y, et al. Three-dimensional MoS2@CNT/RGO network composites for high-performance flexible supercapacitors.Chem A Eur J. 2017;23:3438–3446. 10.1002/chem.201605465 28078805

[33] Huang K-J, Wang L, Zhang J-Z, et al. One-step preparation of layered molybdenum disulfide/multi-walled carbon nanotube composites for enhanced performance supercapacitor. Energy. 2014;67:234–240.

[34] Li J, Shi Q, Shao Y, et al. Cladding nanostructured AgNWs-MoS2 electrode material for high-rate and long-life transparent in-plane micro-supercapacitor. Energy Storage Mater. 2019;16:212–219.

[35] Huang K-J, Wang L, Liu Y-J, et al. Layered MoS2–graphene composites for supercapacitor applications with enhanced capacitive performance. Int J Hydrogen Energy. 2013;38:14027–14034.

[36] Thangappan R, Kalaiselvam S, Elayaperumal A, et al. Graphene decorated with MoS2 nanosheets: a synergetic energy storage composite electrode for supercapacitor applications. Dalton Trans. 2016;45:2637–2646. 26732466 10.1039/c5dt04832j

[37] Huang K-J, Wang L, Liu Y-J, et al. Synthesis of polyaniline/2-dimensional graphene analog MoS2 composites for high-performance supercapacitor. Electrochim Acta. 2013;109:587–594.

[38] Soon JM, Loh KP. Electrochemical double-layer capacitance of {MoS}[sub 2] nanowall films. Electrochem Solid-State Lett. 2007;10:A250.

[39] Lin Z, Liu Y, Halim U, et al. Solution-processable 2D semiconductors for high-performance large-area electronics. Nature. 2018;562:254–258. 30283139 10.1038/s41586-018-0574-4

[40] Raman V, Cho Y-H, Park J-H, et al. Impact of low temperature plasma annealing for flexible, transparent and conductive ITO/PEDOT:PSS composite electrode. J Ind Eng Chem. 2020;93:423–429.

[41] Anandalakshmi K, Venugobal J, Ramasamy V. Characterization of silver nanoparticles by green synthesis method using Pedalium murex leaf extract and their antibacterial activity. Appl Nanosci. 2016;6:399–408.

[42] Yang L, Cui X, Zhang J, et al. Lattice strain effects on the optical properties of MoS2 nanosheets. Sci Rep. 2014;4:1–7. 10.1038/srep05649 PMC4090623 25008782

[43] Raman V, Cho YH, Park JH, et al. Impact of low temperature plasma annealing for flexible, transparent and conductive ITO/PEDOT:PSS composite electrode. J Ind Eng Chem. 2021;93:423–429.

[44] Lee J, Lee P, Lee HB, et al. Room-temperature nanosoldering of a very long metal nanowire network by conducting-polymer-assisted joining for a flexible touch-panel application. Adv Funct Mater. 2013;23:4171–4176.

[45] Da Silva LM, Cesar R, Moreira CMR, et al. Reviewing the fundamentals of supercapacitors and the difficulties involving the analysis of the electrochemical findings obtained for porous electrode materials. Energy Storage Mater. 2020;27:555–590.

[46] Getachew T, Mehretie S, Yip H-L, et al. Roll-to-roll printed high voltage supercapattery in lead-contaminated aqueous electrolyte. Phys Chem Chem Phys. 2020;22:5597–5603. 32100761 10.1039/c9cp06730b

[47] Liu L, Choi S. PEDOT:PSS/MnO2/CNT ternary nanocomposite anodes for supercapacitive energy storage in cyanobacterial biophotovoltaics.ACS Appl Energy Mater. 2020;3:10224–10233.

[48] Randviir EP, Banks CE. Electrochemical impedance spectroscopy: an overview of bioanalytical applications. Anal Methods. 2013;5:1098–1115. 10.1039/d2ay00970f 36342043

[49] Nikam RD, Kwak M, Lee J, et al. Near ideal synaptic functionalities in Li ion synaptic transistor using Li3POxSex electrolyte with high ionic conductivity. Sci Rep. 2019;9:1–11. 31827190 10.1038/s41598-019-55310-8 PMC6906484

[50] Seo KW, Noh YJ, Na SI, et al. Random mesh-like Ag networks prepared via self-assembled Ag nanoparticles for ITO-free flexible organic solar cells. Sol Energy Mater Sol Cells. 2016;155:51–59.

[51] Chao Y, Ge Y, Chen Z, et al. One-Pot hydrothermal synthesis of solution-processable MoS 2 /PEDOT:PSS composites for high-performance supercapacitors. ACS Appl Mater Interfaces. 2021;13:7285–7296. 10.1021/acsami.0c21439 33528246

[52] Wang J, Polleux J, Lim J, et al. Pseudocapacitive contributions to electrochemical energy storage in TiO 2 (anatase) nanoparticles. J Phys Chem C. 2007;111:14925–14931.

[53] Wang Y, Song Y, Xia Y. Electrochemical capacitors: mechanism, materials, systems, characterization and applications. Chem Soc Rev. 2016;45:5925–5950. 27545205 10.1039/c5cs00580a

[54] Zhang C (John), Anasori B, Seral-Ascaso A, et al. Transparent, flexible, and conductive 2D titanium carbide (MXene) films with high volumetric capacitance. Adv Mater. 2017;29:1702678. 10.1002/adma.201702678 28741695

[55] Chen T, Peng H, Durstock M, et al. High-performance transparent and stretchable all-solid supercapacitors based on highly aligned carbon nanotube sheets. Sci Rep. 2014;4:1–7. 10.1038/srep03612 PMC3885879 24402400

[56] Lee K, Lee H, Shin Y, et al. Highly transparent and flexible supercapacitors using graphene-graphene quantum dots chelate. Nano Energy. 2016;26:746–754.

[57] Liu XY, Gao YQ, Yang GW. A flexible{,} transparent and super-long-life supercapacitor based on ultrafine Co3O4 nanocrystal electrodes. Nanoscale. 2016;8:4227–4235. 26838964 10.1039/c5nr09145d

[58] Haacke G. New figure of merit for transparent conductors. J Appl Phys. 1976;47:4086–4089.

[59] Lee JE, Kim HK. Self-cleanable, waterproof, transparent, and flexible Ag networks covered by hydrophobic polytetrafluoroethylene for multi-functional flexible thin film heaters. Sci Rep. 2019;9:1–11. 31723210 10.1038/s41598-019-53243-w PMC6853968

